# Hautabszess verursacht durch *Actinomyces radingae*

**DOI:** 10.1007/s00105-024-05313-y

**Published:** 2024-02-15

**Authors:** Miodrag Davidovic, Miriam Eva Ecker, Anne Kathrin Lößlein, Kilian Eyerich, Christoph Mathis Schempp

**Affiliations:** 1 Klinik für Dermatologie und Venerologie, Universitätsklinikum Freiburg, Freiburg, Deutschland; 2 Institut für Medizinische Mikrobiologie und Hygiene, Universitätsklinikum Freiburg, Freiburg, Deutschland; 3 Klinik für Dermatologie und Venerologie, Universitätsklinikum Freiburg, Medizinische Fakultät, Albert-Ludwigs-Universität Freiburg, Hauptstr. 7, 79104 Freiburg, Deutschland

**Keywords:** Hautinfektion, Seltene Erreger, Molekulare Diagnostik, MALDI-TOF-Massenspektrometrie, 16s-rDNA-Sequenzierung, Skin infection, Rare pathogens, Molecular diagnostics, MALDI-TOF mass spectrometry, 16s rDNA sequencing

## Abstract

Wir berichten über einen 77-jährigen Patienten, bei dem aus einem Abszess *Actinomyces radingae* isoliert wurde. Nach antibiogrammgerechter Therapie mit Amoxicillin/Clavulansäure über 6 Wochen kam es zu einer Abheilung der Infektion. *A. radingae* ist ein seltener Erreger von Haut- und Weichteilinfektionen. Wie bei anderen *Actinomyces*-Infektionen sind aufgrund des chronischen Verlaufs und der langen Therapiedauer eine frühzeitige Erregeridentifizierung und zielgerichtete antibiotische Therapie entscheidend für die optimale Sanierung der Infektion. In der Regel ist *A. radingae* sensibel auf β‑Lactam-Antibiotika.

## Abstract (en)

We report a 77-year-old man with a skin abscess caused by *Actinomyces rading*ae. Targeted antibiotic therapy with amoxicillin/clavulanic acid for 6 weeks resulted in clearing of the infection. *A. radingae* is a rare pathogenic agent of skin and soft tissue infections. As with other *Actinomyces *infections, the early identification of the pathogen and specific antibiotic therapy is crucial for successful resolution of the infection because of the chronic course and the long treatment time needed. Usually, *A. radingae* is sensitive to β‑lactam antibiotics.

## Anamnese

Wir berichten über einen 77-jährigen Patienten, der sich mit einem seit 5 Tagen bestehenden Abszess am Rücken in unserer Ambulanz vorstellte. An der genannten Lokalisation sei vor 3 Jahren, fraglich nach Verletzung bei der Gartenarbeit, ein subkutaner Knoten entstanden, der bisher keine Beschwerden verursacht habe. Der Abszess habe sich nun spontan eröffnet, und eine große Menge Eiter habe sich entleert.

## Befund

**Abb. 1:**
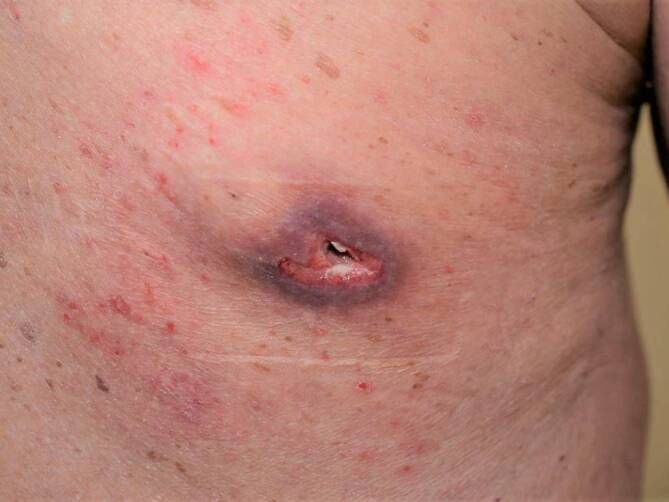
Ansicht des ca. 6 × 4 cm großen lividen Abszesses am oberen Rücken, aus dem sich ein kornartiges Sekret und Eiter entleert. Der Patient erteilte sein schriftliches Einverständnis zur Veröffentlichung der Abbildung

Bei der Vorstellung in unserer Klinik zeigte sich ein ca. 6 × 4 cm großer Knoten mit einem ca. 1 cm großen Orificium, aus dem sich auf seitlichen Druck ein kornartiges mit Eiter und Blut vermischtes Sekret entleerte (Abb. 1). Wir entnahmen Abstriche vom Abszessgrund und begannen eine antibiotische Therapie, zunächst mit Clindamycin 600 mg 3‑mal täglich p.o. Die Wunde wurde mit Povidon-Jod-Salbe und sterilem Pflaster behandelt.

## Diagnostik

**Abb. 2:**
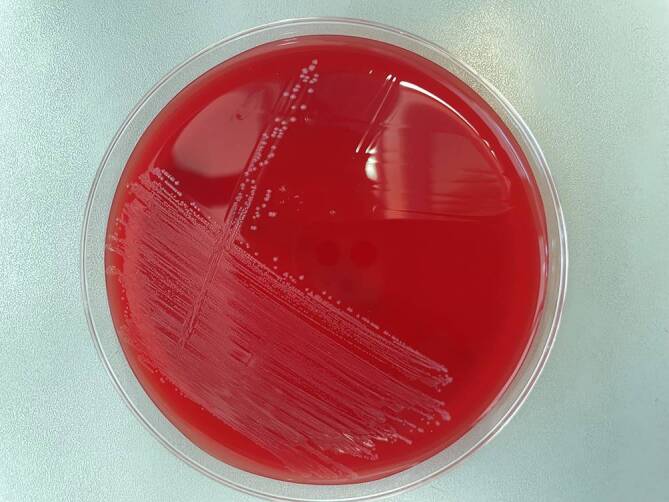
Feine, weißliche bis transparente Kolonien von *A.* *radingae* nach 5 Tagen Inkubation auf einem auf Columbia-Blutagar mit 5 % Schafsblut

Die Anlage des Wundabstrichs erfolgte auf Columbia-Blutagar mit 5 % Schafsblut (Oxoid), MacConkey-Agar und Hefeextrakt-Cystein-Blutagar (HCB). Zusätzlich wurde eine Anreicherungskultur in einer Herz-Hirn-Bouillon angelegt. Die HCB-Kultur wurde für 48 h bei 37 °C ohne CO_2_ anaerob bebrütet. Die anaerobe Atmosphäre wurde mittels GENbox anaer Packs (bioMérieux, Nürtingen, Deutschland) generiert. Die aeroben Kulturen wurden bei 37 °C in 5 % CO_2_ bebrütet und täglich beurteilt. Drei bis 5 Tage nach Anlage zeigte sich aerob das Wachstum feiner Kolonien (Abb. 2). Fünf Tage nach Anlage konnte die Identifizierung des Erregers mittels MALDI-TOF-MS (Bruker Daltonics, Billerica, MA, U.S.A.) erfolgen. MALDI-TOF-MS ist eine Methode der Massenanalyse von chemischen Verbindungen. Das Verfahren kombiniert die Matrix-assistierte Laser-Desorption-Ionisierung (MALDI) mit der Flugzeitanalyse (engl. „time of flight“ [TOF]) freigesetzter Ionen zur Massenspektrometrie (MS). Der Abgleich mit der MS-Datenbank ergab *Actinomyces radingae*. Zur Bestätigung der Massenspektrometrie wurde das als *A. radingae *identifizierte Isolat auf 16s-rDNA-Ebene sequenziert. Hierzu erfolgten die DNA-Präparation einer Einzelkolonie und die Amplifikation der 16S-Region mittels der Primer (5′ → 3′) TPU1 AGA GTT TGA TC[C/A] TGG CTC AG und RTU4 TAC CAG GGT ATC TAA TCC TGT T. Die PCR-Produkte wurden mittels Agarosegelelektrophorese nachgewiesen. Nach Aufreinigung erfolgte die Sequenzierung nach Sanger bei dem externen Anbieter Eurofins Genomics. Es wurden 2 Sequenzierungen jeweils mit dem TPU1- (1) und dem RTU4- (2) Primer durchgeführt. Die sequenzierten Produkte hatten eine Länge von 714 Basenpaaren (1) und 730 Basenpaaren (2). Beim Abgleich der Sequenzen ergab sich eine Übereinstimmung mit *A. radingae* in der Sepsitest™ BLAST®-Datenbank von Molzym (Bremen, Deutschland) (Version 34) mit einer Sequenzidentität von 98,7 und 98,5 % über eine Alignmentlänge von 707 und 754 Basenpaaren (bp). Beim Abgleich der NCBI-Datenbank BLAST® konnte das Genus *Actinomyces *ebenfalls bestätigt werden. Die minimalen Hemmkonzentrationen (MHKs) wurden auf Brucella-Blut-Agar aus einer Suspension McFarland‑1 in Pepton-Bouillon mittels Gradientendiffusionstest (Liofilchem, Roseto degli Abruzzi, Italien, MIC Test-Strips) bestimmt. Die Ergebnisse der Resistenztestung sind in Tab. 1 aufgeführt (Tab. 1). Zudem wurde aus dem Abstrich neben *A. radingae* auch *Peptinophilus harei* angezüchtet. *P. harei* ist ein grampositives, obligat anaerobes Bakterium. Anaerobier finden sich meistens in Mischinfektionen, v. a. in Zusammenhang mit Aktinomykosen.

**Tab. 1:** Ergebnis der Resistenztestung von *A.* *radingae*. Die Grenzwerte zur Beurteilung der Empfindlichkeit wurden der EUCAST Breakpoint-Tabelle für anaerobe Bakterien, Version 11.0 entnommen

	MHK in mg/l	Bewertung
Penicillin	0,023	S
Amoxicillin/Clavulansäure	< 0,016	S
Clindamycin	> 256	R
Vancomycin	0,25	S

*MHK* minimale Hemmkonzentration, *S* sensibel, *R* resistent

## Therapie und Verlauf

Nach Vorliegen des Antibiogramms wurde die antibiotische Therapie bei Resistenz von *A. radingae* gegenüber Clindamycin und wegen Nachweis von *P. harei* auf Amoxicillin/Clavulansäure 875/125 3‑mal täglich für 6 Wochen umgestellt. Der Anaerobier *P. harei* war auf alle getesteten Antibiotika sensibel. Die Therapiedauer betrug nach Empfehlung der Abteilung für Infektiologie insgesamt 6 Wochen. Hierunter kam es zu einer Rückbildung der lokalen Rötung und Schwellung. Die verbleibende zystenartige indurierte Abszesshöhle wurde gegen Ende der Antibiotikatherapie in lokaler Betäubung komplett exzidiert. Es kam zu einer komplikationslosen Abheilung des Befundes.

## Diskussion

Aktinomyzeten sind grampositive, verzweigte Stäbchen, die als kulturell anspruchsvolle, fakultativ anaerobe und langsam wachsende Erreger gelten [6]. Sie können physiologisch auf der Schleimhaut vorkommen, werden aber auch als Erreger von Haut- und Weichteilinfektionen beschrieben [6]. Ein typisches, wenn auch seltenes Krankheitsbild ist die Aktinomykose, eine chronisch verlaufende aerob-anaerobe Mischinfektion, deren Diagnostik eine Herausforderung darstellen kann. Verschiedene Organe wie Lunge oder auch kraniozervikale Weichteile können betroffen sein [2, 5]. In der Dermatologie sind v. a. kraniozervikale Weichteilinfektionen mit *Actinomyces israelii* bekannt [6]. Wir beschreiben hier eine durch *Actinomyces radingae *(*A. radingae*) verursachte Haut- und Weichteilinfektion am Oberkörper.

Bei *A. radingae *handelt es sich um eine Spezies, die erstmalig im Jahr 1995 beschrieben wurde [10]. Die Spezies *A. radingae* ist Teil des Mikrobioms der Haut und des Urogenitaltrakts [6]. Bisher sind insgesamt nur wenige Fälle einer Infektion des Menschen mit *A. radingae* beschrieben worden. Hierbei handelt es sich meist um kutane Abszesse und Weichteilinfektionen am Oberkörper [1, 3, 7, 9]. *A. radingae* wurde kürzlich auch erstmals als Erreger eines Protheseninfektes beschrieben [4]. In Abszessen lassen sich neben *A. radingae* häufig auch anaerob wachsende Erreger nachweisen [7]. Auch beim hier vorgestellten Fall konnte in der Abszesshöhle neben *A. radingae* ein Anaerobier, *P. harei*, nachgewiesen werden.

Die Aktinomykose ist eine langsam progrediente, meist indolente, chronische granulomatöse Infektion [8]. Viele Aktinomyzeten sind Bestandteil der kommensalen Flora der Mundschleimhaut, des Pharynx, des Gastrointestinal- und Urogenitaltrakts. Zu einer Infektion und Entfaltung des pathogenen Charakters der Keime kommt es oft durch Autoinokulation nach Trauma wie Zahnextraktion, intravenöse oder intraartikuläre Therapien, Hautverletzungen, Aspiration oder hämatogene Ausbreitung. Beim hier vorgestellten Patienten besteht ein fraglicher Zusammenhang der Infektion mit einer Verletzung bei der Gartenarbeit, durch die der Erreger eventuell in das Gewebe eindringen konnte. Der Nachweis von Aktinomyzeten ist in der Regel aufgrund des langsamen Wachstums, der anspruchsvollen Kulturbedingungen und der oft negativen Kulturergebnisse (v. a. bei Probenentnahme nach Beginn einer antibiotischen Therapie) schwierig [8]. Da *Actinomyces*-Arten sehr langsam wachsen, ist es wichtig, dass auch eine längere Bebrütung des Untersuchungsmaterials im Labor erfolgt. Deswegen ist es für das Labor hilfreich, wenn die Verdachtsdiagnose Aktinomykose genannt wird. Erst seit der Entwicklung neuer Nachweismethoden wie MALDI-TOF oder 16s-RNA-Sequenzierung ist eine schnelle und präzise Erregerbestimmung möglich.

Bei größenprogredienten, indolenten, knotigen Läsionen und Allgemeinsymptomen wie febrilen oder subfebrilen Temperaturen kommen differenzialdiagnostisch eine kutane fistulierende Tuberkulose, syphilitische Gummen, Sporotrichose, Lymphogranuloma venereum, Nokardiose, maligne Tumoren oder kutane Metastasen infrage. Diagnostisch können dann Sonographie, CT- oder MRT-Aufnahmen hilfreich sein [6]. Die Therapie der Wahl bei *Actinomyces*-Infektionen stellt Penicillin G (intravenös) dar. Im hier vorgestellten Fall wurde wegen Nachweis des Anaerobiers *Peptinophilus harei* aufgrund des breiteren Wirkspektrums über 6 Wochen mit Amoxicillin/Clavulansäure behandelt. Auch die in der Literatur beschriebenen kutanen Infektionen mit *A. radingae* als Erreger wurden ebenfalls erfolgreich mit β‑Lactam-Antibiotika therapiert [6]. Die Behandlung einer Aktinomykose kann sich unter Umständen aber auch sehr langwierig gestalten und eine Therapiedauer mit hoch dosierten Antibiotika von bis zu 1 Jahr erfordern [6].

## Fazit für die Praxis

*Actinomyces radingae* ist ein bisher selten beschriebener Erreger von Haut- und Weichteilinfektionen, v. a. am Oberkörper. Wie bei anderen *Actinomyces*-Infektionen sind aufgrund des chronischen Verlaufs und der langen Therapiedauer eine frühzeitige Erregeridentifizierung und zielgerichtete antibiotische Therapie entscheidend für die optimale Sanierung der Infektion. In der Regel ist *A. radingae* sensibel auf β‑Lactam-Antibiotika.
